# Quadriceps muscle architecture ultrasonography of individuals with type 2 diabetes: Reliability and applicability

**DOI:** 10.1371/journal.pone.0205724

**Published:** 2018-10-18

**Authors:** Camilla Rodrigues de Souza Silva, André dos Santos Costa, Taciano Rocha, Diogo Arruda Martins de Lima, Tamires do Nascimento, Sílvia Regina Arruda de Moraes

**Affiliations:** 1 Programa de Pós-Graduação em Neuropsiquiatria e Ciências do Comportamento, Universidade Federal de Pernambuco, Recife, Brasil; 2 Departamento de Educação Física,Universidade Federal de Pernambuco, Recife, Brasil; 3 Programa de Pós-Graduação em Fisioterapia, Universidade Federal do Rio Grande do Norte, Natal, Brasil; 4 Departamento de Fisioterapia, Universidade Federal de Pernambuco, Recife, Brasil; 5 Departamento de Anatomia, Universidade Federal de Pernambuco, Recife, Brasil; University of Maiduguri College of Medical Sciences, NIGERIA

## Abstract

Muscle architecture parameters performed using ultrasound serve as an aid to monitor muscle changes derived from diseases, however there are no studies that determine the reliability and applicability of this evaluation in individuals with type 2 diabetes (DM2). Three raters captured three images of measurements of thickness of the rectus femoris (RF), vastus intermedius and anterior quadriceps, RF muscle cross-sectional area, RF pennation angle in 17 individuals with DM2 above 50 and sedentary. Intra and inter-raters analysis showed reliability from high to very high for the three raters (ICC> 0.87), except for the RF pennation angle with moderate to low intra-raters (ICC = 0.58, 0.48, 0.51), and high inter-rater reliability (ICC = 0.70). Ultrasound measurements of quadriceps muscles showed high to very high intra and inter-raters reliability, thus allowing its use to monitor muscle changes provoked by diabetes or interventions in individuals with DM2.

## Introduction

Diabetes *mellitus* facilitates the installation and development of chronic complications [1], such as diabetic neuropathy [2, 3], and sarcopenia [4]. People with diabetes are more likely to suffer accelerated loss of mass and muscle strength over time, particularly in the lower extremities [5, 6] which is related to the increased risk of mortality in individuals with type 2 diabetes [7].

Interventions to augment lower limb muscle strength have been suggested to enhance mobility and quality of life of patients with type 2 diabetes [8]. In this sense, physical training has been proven positive in this population, minimizing muscular deficits while allowing meaningful changes in body composition, such as fat loss and increased lean body mass with improvements in muscle strength [9, 10].

One parameter used to assess the function and morphology of the muscle is the analysis of skeletal muscle architecture, defined as the geometric arrangement of muscle fibers [11] and that can be evaluated in a non-invasive and low-cost way through ultrasound (US) [12]. Muscle thickness and pennation angle are critical parameters when assessing muscle function during human movements [13]. Muscle thickness is linked with strength [14], whereas pennation angle is with the efficient transmission of muscle fiber force to the tendon [15].

Thus, it is probable that assessing parameters of muscular architecture performed using US will serve as aid to monitor muscle modifications derived from illness and responses to interventions proposed to these individuals. However, studies involving assessment regarding reliability of measurement of these parameters in lower limbs were performed in healthy individuals, or in neuromuscular or critically ill patients [16, 17] though, so far, such claim is nonexistent in the literature as to individuals with type 2 diabetes. Therefore, the purpose of this study was to use US technique and measure the thicknesses of RF, vastus intermedius (VI) and anterior quadriceps, including the cross-sectional area and pennate angle of the RF to underpin reliability values of these measurements in individuals with type 2 diabetes.

## Methods

### Design and participants

It is a cross-sectional study made between November 2016—April 2017. Were recruited participants volunteers with type 2 diabetes with diabetic neuropathy, over 50 years of age, sedentary and with no history of lower limbs osteomioarticular injuries. They signed a free and clear consent form prior to the study. Assessments were performed in the Laboratory of Ultrasonography of the Complex of Laboratories Prof. José César de Albuquerque Farias of the Department of Physical Education of the Federal University of Pernambuco. This study is in conformity with Resolution 466/12 of CNS / Brazil, the Declaration of Helsinki for research involving human beings, and was previously approved by the Committee of Ethics in Research Committee of the Federal University of Pernambuco under the protocol number CAAE: 45169515.7.0000.5208.

### Assessment with ultrasound

The muscle assessment was performed using images in B mode obtained with an ultrasound device LOGIQ P5 (GE Medical Systems Ultrasound & Primary Care Diagnostics, Milwaukee, WI, USA); as well as a transducer with a sampling frequency between 10–13 MHz and with a viewing area of 4 centimeters.

The minimal contact pressure when takin measurements was ensured by placing the transductor on the right place, exerting enough pressure to create a good image at the screen, then the evaluator released the pressure very slowly until loose the image at the screen but before losing contact with the skin. The time lapse (US device) was used to get back “in time” and assure the best image with less pressure possible. This could be repeated until a satisfactory association between less pressure and good image quality.

**Fig 1:**
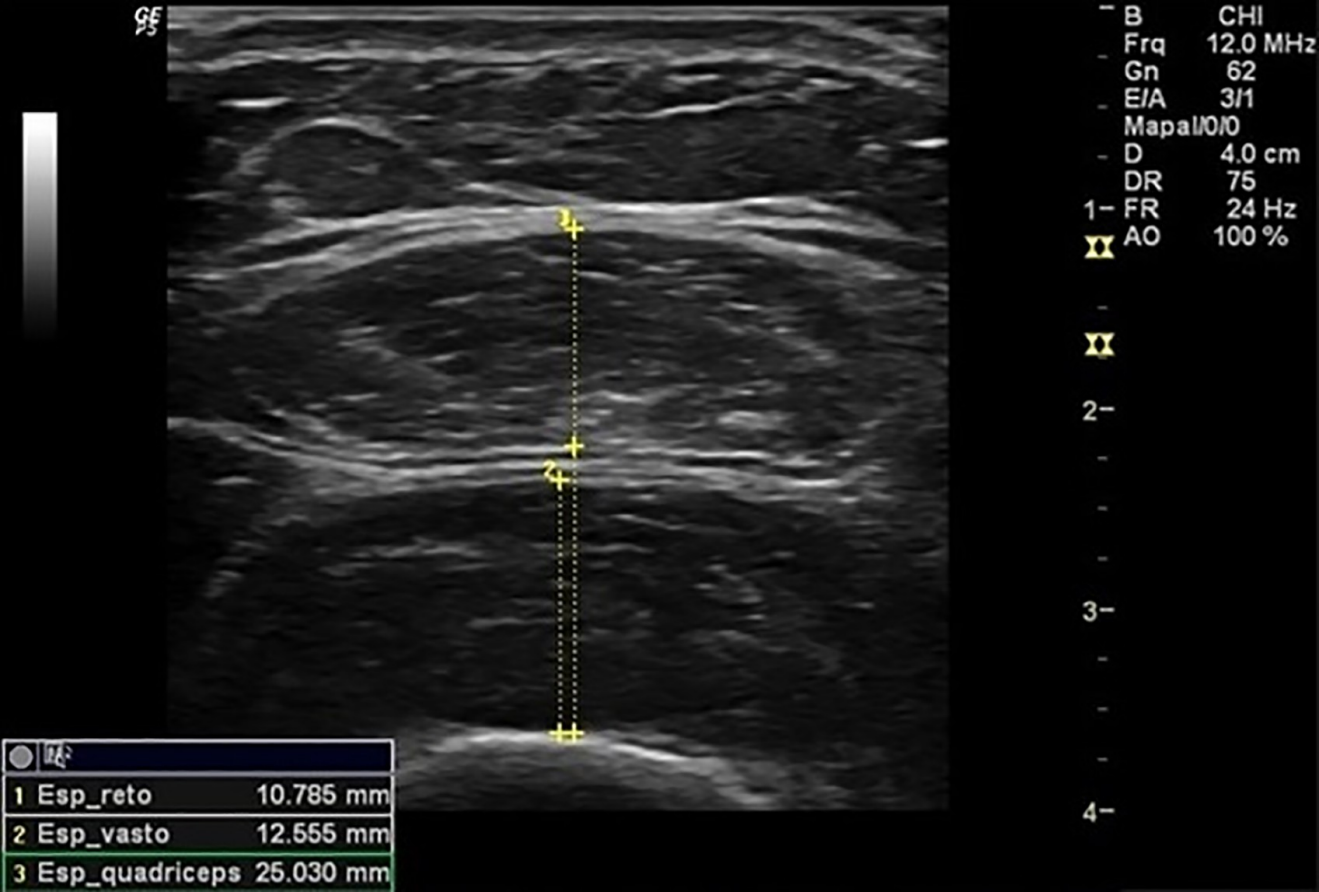
Thickness measurements. Measurements of thickness of rectus, thickness of vastus intermedius and thickness of anterior quadríceps.

**Fig 2:**
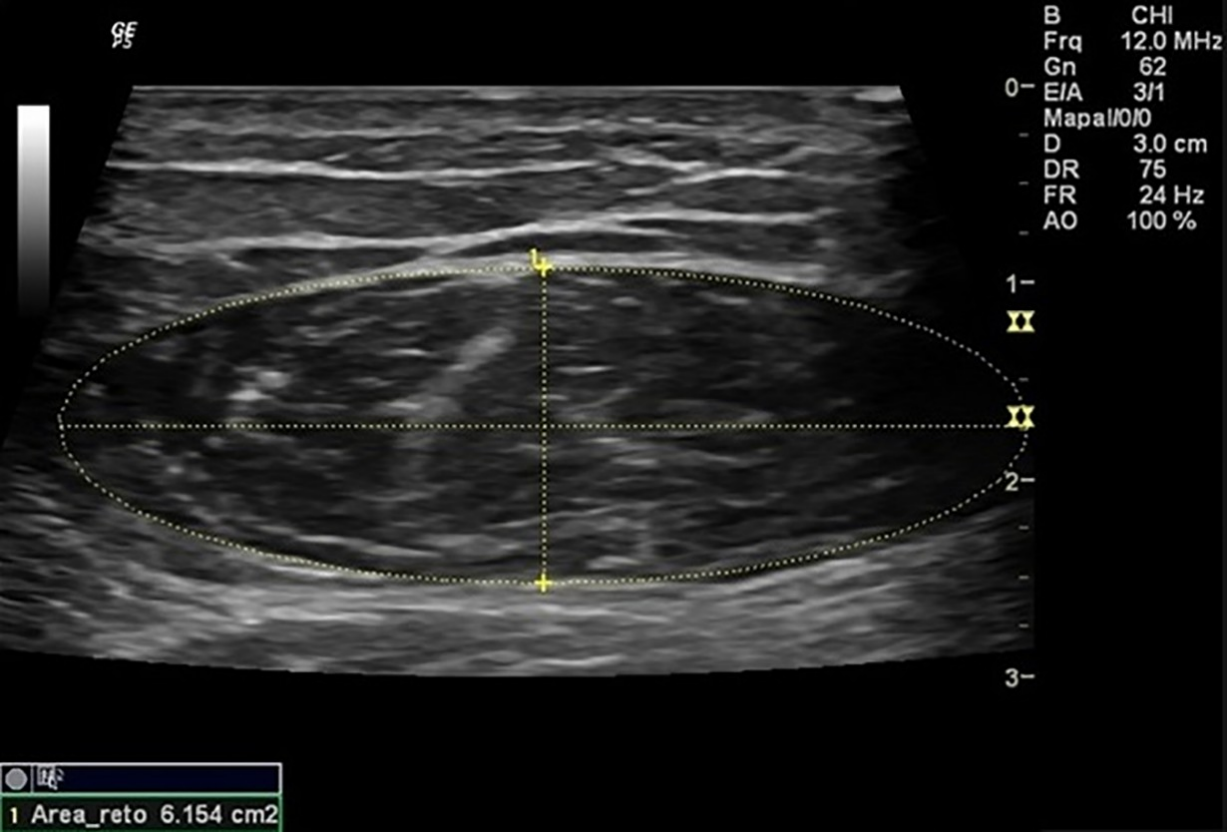
Rectus femoris muscle cross-sectional area. Measurement of rectus femoris muscle cross-sectional area.

**Fig 3:**
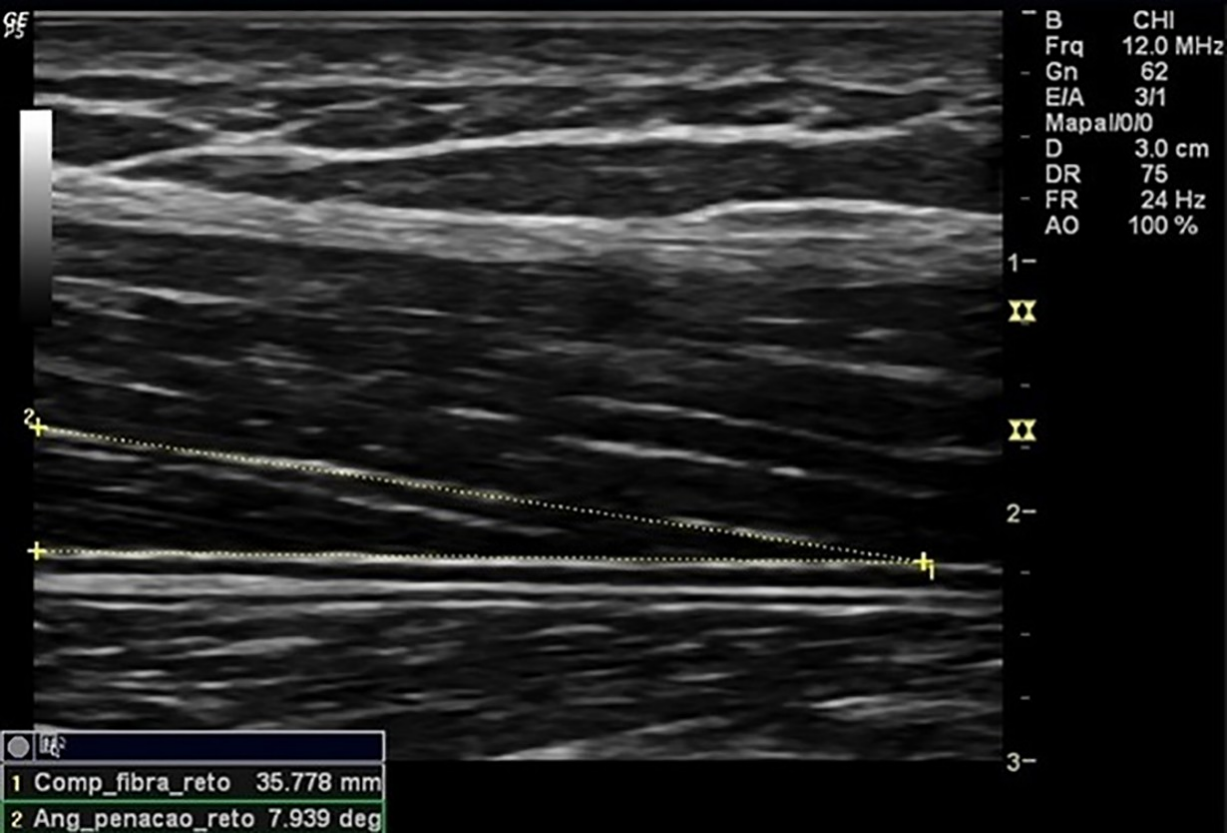
Pennation angle of the rectus femoris muscle. Measurement of rectus femoris pennation angle.

Were assessed the thickness of RF, the thickness of VI, the thickness of anterior quadriceps (Fig 1), the RF muscle cross-sectional area (Fig 2), and the pennation angle of the RF (Fig 3). Assessments were performed during visit on each participant dominant limb. Participants were instructed to hydrate normally and not engage in any exercise session or intense physical activity within 72 hours prior to assessments.

The participant remained in the supine position with 10° knee flexion to capture images, and the transducer positioned at 50% of the distance between the greater trochanter and the interarticular line of the knee, transversely to the muscle for thickness measurements and cross-sectional area, and longitudinally for the measurement of pen angle [18]. Participant´s muscles had to be completely relaxed during measurements, so participants were oriented to perform this. Their placement with 10° knee flexion helped to assure this muscle “inactivation”. Muscle contractions are observed by Muscle US imaging, real time. Since our assessment took some time to be done, we would be able to see if the volunteer made any contractions during the assessment.

The RF thickness was set as the linear distance between the two superficial and internal aponeuroses of the RF muscle; thickness of VI as the linear distance between the superficial aponeurosis of the VI muscle and the surface of the femur; thickness of anterior quadriceps as the linear distance between the superficial aponeurosis of the RF muscle and the femur surface; the pennation angle of the RF determined at the point of intersection between the muscle fascicles of the rectus femoris and the internal aponeurosis, through the angle formed between both structures. The RF muscle cross-sectional area was defined by the area of the ellipse formed between the two superficial and internal aponeuroses of the RF muscle in the expanded image mode.

Images were taken by each of the previously trained 3 raters who captured 3 images for each assessed parameter, resulting in an average of 3 measurements of each rater of each assessed parameter. The images were assessed using software in-built in the Ultrasound Device, real time.

Data sheet of the muscular architecture parameters measurements obtained by each rater for this study is available as supporting information; see S1 File.

### Statistical analysis

Data were analyzed using SPSS 20.0 (IBM Corp., Chicago, IL, USA). The normality of the data of each variable was analyzed with the Shapiro-Wilk test and the data were described as mean and standard deviation. The limit of statistical significance was set at P <0.05.

For intra-rater reliability analysis, the coefficient of interclass correlation and confidence intervals (ICC and 95% CI), including the coefficient of variation (CV), which was calculated from the division of the standard deviation (SD) by the mean value of the three measures (CV = [SD/mean]*100). The inter-rater analysis considered the comparison between the means of each rater, which provided a value of ICC (95% CI) and an average CV calculated from the mean of the CV of each individual, followed by the calculation of the standard error measurement (SEM) resulting from the ratio between the SD of the differences obtained between the measures of the raters and the square root of the number two (SEM = SD/√2) [19], thus enabling computation of each variable minimal detectable difference (MDD) as MDD = 1,96 x SEM x √2 [20].

For ICC classification values between 0.00–0.25 were considered as unreliability; those between 0.26–0.49 as low reliability; the ones between 0.50–0.69 as moderate reliability; between 0.70–0, 89 as high reliability, and between 0.90–1.00 as very high reliability [21].

## Results

Seventeen volunteers participated in the study (11 women: 58.9 ± 4.6 years, 72.2 ± 11.3 kg, 157 ± 4 cm, and 6 men: 61.2 ± 4.5 years, 74.7 ± 9.6 kg, 167.6 ± 7.9 cm).

The Shapiro-Wilk test showed a normal distribution of inter- and intra-rater measurements. Detailed data of the reliability analysis are shown in Table 1.

**Table 1:** Descriptive values of intra and inter-rater reliability analysis.

		Mean ± SD	ICC (95%IC)	SEM	CV (%)	MDD
**RF thickness (mm)**	R1	12,12 ± 2,56	0,96 (0,91 a 0,98)	1,48	3,35	
	R2	13,30 ± 2,76	0,97 (0,93 a 0,98)	1,37	3,0	
	R3	12,75 ± 2,57	0,95 (0,90 a 0,98)	1,65	3,82	
	Inter	12,74 ± 2,49	0,93 (0,85 a 0,97)	1,98	6,70	5,48
**VI thickness (mm)**	R1	12,61 ± 3,22	0,99 (0,98 a 0,99)	0,86	2,07	
	R2	12,33 ± 2,66	0,98 (0,95 a 0,99)	1,14	2,66	
	R3	12,29 ± 2,97	0,97 (0,95 a 0,99)	1,27	2,77	
	Inter	12,39 ± 2,76	0,92 (0,84 a 0,97)	2,24	7,66	6,21
**Anterior quadriceps (mm)**	R1	26,06 ± 4,93	0,98 (0,96 a 0,99)	1,81	1,70	
	R2	27,13 ± 4,02	0,97 (0,95 a 0,99)	1,77	1,80	
	R3	26,25 ± 4,56	0,87 (0,74 a 0,94)	4,50	3,21	
	Inter	26,43 ± 4,28	0,93 (0,85 a 0,97)	3,30	6,18	9,14
**RF cross-sectional area (cm**^**2**^**)**	R1	4,37 ± 1,52	0,96 (0,93 a 0,98)	0,81	4,80	
	R2	4,50 ± 1,68	0,96 (0,93 a 0,98)	0,88	5,46	
	R3	4,73 ± 1,58	0,98 (0,96 a 0,99)	0,47	3,36	
	Inter	4,53 ± 1,50	0,94 (0,87 a 0,98)	1,07	12,16	2,97
**RF pennation angle (degrees)**	R1	8,57 ± 1,99	0,58 (0,30 a 0,80)	3,86	15,97	
	R2	11,92 ± 5,52	0,48 (0,20 a 0,74)	5,41	18,44	
	R3	8,88 ± 2,38	0,51 (0,22 a 0,76)	5,07	21,68	
	Inter	8,76 ± 1,78	0,70 (0,32 a 0,88)	2,93	15,82	8,12

RF = rectus femoris; VI = vastus intermedius; R1 = rater 1; R2 = rater 2; R3 = rater 3; SD = standard deviation; ICC = intra-class correlation coefficient; IC = confidence interval; SEM = standard error of measurement; CV = coefficient of variation; MDD = minimal detectable difference.

The intra-rater analyzes showed very high reliability by the three raters (ICC> 0.9), except for the thickness of anterior quadriceps parameter measured by the rater 2 that presented a high reliability (ICC = 0.87), and for the pennation angle of the RF parameter showing moderate reliability by raters 1 and 3 (ICC = 0.58, 0.48, 0.51), and low reliability by rater 2 (ICC = 0.48). The highest CV and MDD were observed in the pennation angle of the RF variable.

In the inter-analyzer analysis, very high reliability was observed in all assessed parameters (ICC> 0.9), except for the pennation angle of the RF parameter showing a high reliability (ICC = 0.70). The highest CV was observed in pennation angle of the RF (16%), followed by RF muscle cross-sectional area (12%), while the lowest CV was in the thickness of anterior quadriceps (6%). The highest SEM was observed in the thickness of anterior quadriceps variable (3.3 mm), while the lowest SEM value was observed in the pennation angle of the RF (1.07 mm). MDD values varied directly in proportion to the SEM values, with a maximum value in the thickness of anterior quadriceps variable (9.14 mm), and a minimum value in the pennation angle of the RF variable (2.97 mm).

## Discussion

To our knowledge, this is the first study that assess the reliability of the US in muscular quadriceps architecture measurements of individuals with type 2 diabetes.

Our results showed that the US presented a high to very high reliability for the determination of the parameters of thickness of RF, thickness of VI, thickness of anterior quadriceps, RF muscle cross-sectional area, and pennation angle of the RF muscle in the intra and interrater analysis; which suggests that such muscular architecture parameters obtained via quadriceps US images can be used as reliable measures to assess the size, quality, muscle area, including monitoring possible modifications resulting from nutritional interventions and exercise in the muscular architecture of this diabetic population.

ICC values of muscle thickness (RF, VI, anterior quadriceps) were in a range of 0.87–0.99 for the quadriceps, considering the intra and interrater analysis, which is a high to very high reliability. These results are in agreement with previous studies that assessed the reliability of measurements in the quadriceps muscles of healthy men and young women in intra-rater analysis [13, 22, 23], and critically ill young adults [24], in the same analysis. The highest reliability observed in the present study was for thickness of VI (ICC = 0.99) obtained by rater 1; which is identical to the result observed in the study of Ruas et al [22] that analyzed the same parameter in 10 healthy schoolchildren.

CV and SEM data were not always described by the studies stated above, though one of them [13] presented CV values for RF (2.4%); which were lower than ours (3.0–3.82%). The study by Gomes et al [25] presented the CV for thickness of anterior quadriceps (4.6%) higher than ours (1.70–3.21%) in the intra-rater analysis. SEM values were solely described in the study of Ruas et al [22] (RF = 0.72; VM = 0.73); which were lower than those found in ours (RF = 1,37–1,61; VI = 0.86–1.27). The reasons behind these inconsistences may be related to the muscle changes caused by diabetes or higher age we made use of. Another study reported that age could contribute to differences in quadriceps thickness measurements after testing a cohort that included participants between 17–90 years [26]. In addition, differences in US adjustments may have contributed to the difference in results existing in previous studies.

The RF muscle cross-sectional area also showed very high reliability in this study, both in the intra-rater analysis (ICC = 0.96–0.98) and in the inter-rater (ICC = 0.94). These results substantiate those presented in previous studies in healthy individuals [22, 27–29]. However, CV and SEM values were somewhat lower in the studies that reported these measures when compared to the values found in ours.

Of muscular architecture parameters assessed in this study, only the pennation angle of the RF showed a low to moderate reliability. On the other hand, the study of Ema et al [13] evaluated this same parameter with methodology similar to ours and presented a very high reliability (ICC = 0.95), with a low CV (3.7%). The study, however, was conducted with healthy young individuals.

A clinically important parameter analyzed in the present study was MDD, defined as the minimum detectable difference that should occur between the initial measurement and a subsequent measurement, so that this difference does not correspond to an examiner error, but to a gain or loss generated by an applied intervention [30]. Only the study of Ruas et al [22] submitted this data in the parameters of thickness of RF (2.01mm), thickness of VI (2.03mm), and RF muscle cross-sectional area (0.99cm^2^), in the intra-rater analysis. MDD values found in our study (RF = 5.48mm, VI = 6.21mm, cross-sectional area = 2.97cm^2^) were higher than those of the aforementioned study, although the analysis was performed by inter-raters.

There is a direct effect of diabetes on muscle characteristics and muscle performance. Studies have shown that diabetes causes a reduction in muscle quality of about 17–37% in males and 49–69% in females when compared with healthy individuals [31], and that the characteristics of lower limb muscles, such as strength, power and muscular quality represented 24.3 and 15.1% of the walking speed difference, comparing diabetic and non-diabetic individuals in 4 and 400 m walks, respectively [32].

There is no data yet to show the effect of diabetes on muscle architecture parameters. However, a recent study showed that the application of a resistance training protocol in this population was able to significantly increase the thickness of the muscles of the anterior thigh region, and this change was detected through ultrasonography [33]. The increase in thickness found in the cited article was about 5% of the initial thickness, which is well below the MDD values found in our study (30–50%). Despite this, the individuals of the study presented functional benefits, represented by the increase of muscular strength of lower limbs.

Given the potential degenerative loss in the muscular architecture of this population, MDD values found in our study may not be reached over time in interventions applied in this population. Although, this doesn´t not mean that there were no benefits to the study population. Functional parameters such as strength and walking velocity should also be taken into account in the evaluations, being considered as preponderant factors for the success or failure of suggested interventions.

The search for knowledge of diabetes outcomes and development of new criteria of evaluation for this population underscores the need for further studies that extend this analysis to groups of different ages and levels of physical activity, as well as individuals in different stages of the disease, correlating it with their functional outcomes.

In conclusion, the results showed that, with the exception of pennation angle, the measurements of quadriceps muscle thicknesses (RF, VI, anterior quadriceps) and RF muscle cross-sectional area, quantified through the US, showed very high intra and inter-rater reliability, thus allowing its use for purposes of monitoring muscle changes following interventions such as diet programs, physical training or rehabilitation of individuals with type 2 diabetes.

## Supporting information

S1 File Data sheet of the muscular architecture parameters measurements obtained by each rater. (XLS)
